# Overexpression of Avenin-Like b Proteins in Bread Wheat (*Triticum aestivum* L.) Improves Dough Mixing Properties by Their Incorporation into Glutenin Polymers

**DOI:** 10.1371/journal.pone.0066758

**Published:** 2013-07-02

**Authors:** Fengyun Ma, Miao Li, Tingting Li, Wei Liu, Yunyi Liu, Yin Li, Wei Hu, Qian Zheng, Yaqiong Wang, Kexiu Li, Junli Chang, Mingjie Chen, Guangxiao Yang, Yuesheng Wang, Guangyuan He

**Affiliations:** The Genetic Engineering International Cooperation Base of Chinese Ministry of Science and Technology, Chinese National Center of Plant Gene Research (Wuhan) HUST Part, The Key Laboratory of Molecular Biophysics of Chinese Ministry of Education, College of Life Science and Technology, Huazhong University of Science & Technology(HUST), Wuhan, China; Cankiri Karatekin University, Turkey

## Abstract

Avenin-like b proteins are a small family of wheat storage proteins, each containing 18 or 19 cysteine residues. The role of these proteins, with high numbers of cysteine residues, in determining the functional properties of wheat flour is unclear. In the present study, two transgenic lines of the bread wheat overexpressing *avenin-like b* gene were generated to investigate the effects of Avenin-like b proteins on dough mixing properties. Sodium dodecyl sulfate sedimentation (SDSS) test and Mixograph analysis of these lines demonstrated that overexpression of Avenin-like b proteins in both transgenic wheat lines significantly increased SDSS volume and improved dough elasticity, mixing tolerance and resistance to extension. These changes were associated with the increased proportion of polymeric proteins due to the incorporation of overexpressed Avenin-like b proteins into the glutenin polymers. The results of this study were critical to confirm the hypothesis that Avenin-like b proteins could be integrated into glutenin polymers by inter-chain disulphide bonds, which could help understand the mechanism behind strengthening wheat dough strength.

## Introduction

Wheat is currently an important human food resource that can be processed into a range of foods such as breads, cakes, biscuits, pastas and noodles. The ability of wheat to be processed into such a wide range of foods stems from its particular ability to form cohesive dough. The key component of dough is gluten proteins, which can form a continuous viscoelastic protein network within the dough and confer elasticity and viscosity to the dough [1], [2]. The major gluten proteins are gliadins and glutenins. Monomeric gliadins are considered as single polypeptides with slight differences in molecular weight in the flour [3], [4]. Glutenins, on the other hand, have a wide range of molecular weights and contain high-molecular weight glutenin subunits (HMW-GS) and low-molecular weight glutenin subunits (LMW-GS). These glutenins can form the high molecular mass glutenin polymers and largely contribute to wheat dough strength [5]. The different properties contributed by gliadins and glutenins to dough quality are determined by the difference in the number and position of cysteine residues [6]. In general, gliadins form only intra-chain disulfide bonds [3], [4], while glutenin subunits can form both intra- and inter-chain disulfide bonds [7]. The widely-held view of gluten structure was summarized by Shewry et al. [7] who suggested a structural model for wheat gluten, in which the HMW subunits crosslink with each other in a head-to-tail fashion by inter-chain disulphide bonds and form an ‘elastic backbone’, while LMW subunits crosslink to this backbone basis and form ‘branches’. These elastic backbone formed by HMW subunit with the branches formed by the LMW subunit are the glutenin polymers. Gliadins may also interact with the glutenin polymers by strong covalent and non-covalent forces and contribute to gluten viscosity [2]. In this model, the HMW-GS plays a determinant role in dough strength. This was confirmed in many studies that analyzed the functional properties of near-isogenic [8], [9], [10] and transgenic wheat lines [11], [12], [13], [14], [15], [16], [17]. In addition, several studies also used transgenic wheat lines to demonstrate that LMW glutenin subunits were also important determinants of quality in processed wheat [18], [19], [20], [21].

Further, a new class of wheat storage proteins named Avenin-like proteins was recently identified with its function still unknown. Kan et al. [22] reported two new classes of cDNAs expressed specifically in seed endosperm of wheat and named the encoded proteins a-type and b-type Avenin-like proteins. These Avenin-like proteins were also detected in the glutenin fraction of durum wheat cultivar, Svevo, and considered to be seed storage proteins by the anthors [23], [24]. The distinguishing feature of these seed storage proteins was that they included high numbers of cysteine residues. Avenin-like a-type protein, with a molecular mass of about 18 kDa, contains 14 cysteine residues [22]. Based on the presence of highly conserved cysteine residues in the proteins from different species and its high sequence homology to ‘low-molecular weight gliadin’ monomer [25], [26], [27], it is presumable that cysteine residues of Avenin-like a protein mediate seven intra-chain disulfide bonds. The mature Avenin-like b-type proteins with a molecular mass of about 30 kDa contained 18 or 19 cysteine residues [22]. The variation in the number and position of the cysteine residues in the N- and C-terminal domains of the b-type proteins indicated that some of these cysteine residues could be involved in inter-chain disulphide bonds [22]. This raised the question whether this class of cysteine-rich proteins played a role in determining the functional properties of wheat. In addition, we were also interested in investigating their possible involvement in determining dough strength.

In our previous study, we isolated and characterized twenty-three Avenin-like b genes from wheat and related species and suggested that they were a new class of proteins different from gliadins and glutenins [28]. Expression pattern of the *avenin-like b* gene was seed endosperm-specific and observed between 3 and 22 DPA (days post anthesis) with a peak between 11 and 15 DPA in wheat [28]. This expression pattern was similar to the expression pattern of gliadin and glutenin genes in developing wheat seeds. In a subsequent study, incorporation of purified Avenin-like b proteins, produced *in vitro*, into wheat flour revealed a positive correlation between Avenin-like b proteins and dough strength by 2 g Mixograph tests [29]. In the present study, two transgenic wheat lines overexpressing *avenin-like b* gene were generated by particle bombardment to study the influence of Avenin-like b proteins on the functional properties of wheat flour. The results showed that overexpression of Avenin-like b proteins in transgenic wheat lines had a positive impact on dough Mixograph parameters and provided the evidence for incorporated of Avenin-like b proteins into gluten polymers.

## Materials and Methods

### Plant Material

Bread wheat (*T. aestivum* L.) variety Emai 12 is a poor quality wheat cultivar of the Yangzi River down-central area in China, which is suitable for studying the effect of Avenin-like b proteins on functional properties of wheat flour. They are weak spring-type genotype containing four HMW-GS: Bx7, By8, 1Dx2 and Dy12.

### Vector Construction

Genomic DNA was extracted from wheat leaves (*T. aestivum* L. Zhengmai 9023) using the CTAB method [30]. Primers specific to *avenin-like b* locus were designed from the sequences in the N- and C-terminal domains, which were highly conserved in the b-type proteins. The *avenin-like b* gene was amplified from the genomic DNA of wheat (*T. aestivum* L. Zhengmai 9023) using primers containing the restriction sites *Sal*I and *Bam*HI and having the following sequences: forward primer: 5′-CGCTGTCGACATGAAGGTCTTCATCCTGGCTC-3′ (*Sal*I site underlined); reverse primer: 5′-TCGAGGATCCCTAGCACGCACCACCAGTGTA-3′ (*Bam*HI site underlined). The amplified products were cloned, sequenced and digested with *Sal*I and *Bam*HI, and finally cloned into the *Sal*I/*Bam*HI digested transformation vector pLRPT [31], [32], resulting in its insertion between the endosperm-specific 1Dx5 promoter and the CaMV35S terminator. The recombinant vector named pLRPT-avel was co-bombarded with the plasmid pAHC25 [33] containing the *bar* gene that confers resistance to the herbicide BASTA, and the *uidA* gene that encodes for β-glucuronidase (*GUS*), both under the control of the constitutive maize ubiquitin promoter.

### Genetic Transformation and Plant Regeneration

Immature scutellum explants from the wheat cultivar Emai 12 were co-bombarded with the plasmids pLRPT-avel and pAHC25 at a 2∶1 molar ratio, using the transformation procedure described by Sparks and Jones [34]. Plants were regenerated and selected under the herbicide phosphinotricin (3 mg/ml).

### PCR and Southern Blotting Analysis

Since CaMV35S terminator was the unique sequence in the pLRPT-avel vector which did not have any similarity with the bread wheat genomic DNA, this sequence was used to identify transgenic plants. CaMV35S terminator was amplified using the primer pair: 5′-CGCTGAAATCACCAGTCT-3′ and 5′-TCCTTCCTTCCGTCCACT -3′. The length of the amplified fragment was 417 bp.

Genomic DNA was extracted from leaves of T_0_ transgenic plants by the CTAB method [30] and digested with *Hin*d III and *Bam*H I. Digested genomic (10 µg) and plasmid (5 pg) DNA were separated by electrophoresis in a 0.8% (w/v) agarose gel and transferred onto nylon membrane positively charged, according to the manufacturer’s instructions (Roche). Membrane was hybridized with random-primed generated probes produced using above primers for the CaMV35S terminator sequence (contained in pLRPT-avel). The hybridized probe DNA was detected by exposure to Kodak double-emulsion films.

### Polyclonal Antibody Preparation, Western Blotting and Seed Storage Protein Characterization

The bacterial expression vector pET-32a was used to express Avenin-like b protein in *E.coli*. The primers (forward 5′-CAGTTGGAAACCACATGT-3′, reverse 5′-TAGCACGCACCACCAGT-3′) were designed to amplify only the *avenin-like b* gene coding region (without the signal peptide) from the recombinant vector pLRPT-avel. Construction of the expression vector pET-32a-avel, expression and purification of the recombinant Avenin-like b proteins were carried out following the method described by Chen et al. [29]. Polyclonal antibodies against Avenin-like b proteins were generated by immunizing Japanese white rabbit with the purified and renatured Avenin-like b proteins in a heterologous system.

Total proteins from wheat seeds were extracted from single kernels of transgenic and control lines following the method described by He et al. [14] and separated on a 15% SDS-PAGE. After separation, proteins were electro-blotted onto Polyvinylidene fluoride (PVDF) membrane. Membranes were blocked in 1×TBST (Tris buffered saline plus 0.1% Tween-20) containing 5% NFDM (non-fat dry milk) overnight at 4°C. Primary rabbit anti-avenin-like b protein anti-serum was diluted 1∶2×10^5 ^in TBST/5% BSA and incubated at 25°C for 2.5 h. The membrane was washed four times in TBST and incubated with 1∶1×10^4^ dilution of alkaline phosphatase conjugated goat anti-rabbit secondary antibody at 25°C for 1 h, then detected according to the manufacturer’s instructions. Antibody against the housekeeping protein GAPDH was used to normalize for equal amounts of proteins and to calculate the relative loading volume for each sample. The amounts of Avenin-like b proteins were determined by densitometry analysis of the Western blotting results in three biological replications using a Bio-Rad Quantity One 1-D software version 4.6.2 (Bio-Rad, Hercules, CA).

To characterize storage proteins from each line, gliadins, glutenins and other proteins were sequentially extracted from 100 mg flour from each sample according to DuPont et al. [35]. For densitometry, proteins from 15 flour samples per line were separated on SDS-PAGE and the gliadin, glutenin and albumin/globulin fractions were quantified by densitometry method using a Bio-Rad Quantity One 1-D software version 4.6.2 (Bio-Rad, Hercules, CA). Densitometry was chosen rather than HPLC because of its higher reproducibility in characterization of storage proteins [36].

The soluble proteins and the insoluble proteins from flour for SE-HPLC analysis were extracted following the method described by Tosi et al. [21]. The proportion of total polymeric proteins (%UPP) was determined according to Gupta et al. [37].

### Extraction of Monomeric Subunits, Small Oligomers and Polymers Fractions

Monomeric subunits and small oligomers were extracted with 50 mM of sodium phosphate, pH 6.8, and 0.5% (w/v) SDS (25 µl/mg) using the method described by Tosi et al. [31]. After centrifugation at 10,000 g for 10 min, the supernatant from each sample was recovered and divided into two parts, with β-mercaptoethanol being added to one, to a final concentration of 2.5% (v/v). The proteins remaining in the pellet, consisting almost exclusively of glutenin polymers, were then extracted with the same buffer containing 5% (v/v) β-mercaptoethanol. Reduced and unreduced extracts were analyzed by SDS-PAGE using standard method with 15% separating gels and Western blotting.

### Quality Tests

Seeds from transgenic lines, one non-transgenic line and one non-transformed control line harvested in 2012 were used for analysis of dough mixing properties. Prior to milling, kernel moisture was adjusted to 14% by incubation overnight at room temperature. One hundred grams of seeds per line were milled to flour with a Brabender Quandrumat Junior Mill following AACC method 26–50. The protein contents and moisture contents of flour were measured by near-infrared reflectance spectroscopy (NIRS) method using an Infratec TM1241 Grain Analyzer (Foss North America, Silver Spring, MD). Both the moisture contents and protein contents of transgenic and control lines were averaged from three replications. The water absorption was estimated by Approved Methods (AACC, 1995) using the protein and moisture contents of flour.

Wet gluten content (%), dry gluten content (%), and sodium dodecyl sulfate sedimentation (SDSS) volume (ml) were determined using a Perten 2200 Glutomatic System (Perten Instruments AB, Huddinge, Sweden) and a Brabender Quadrumat Sedimat (Brabender OHG, Duisburg, Germany) and the procedures described by ICC/No. 137/1, 155 & 158 and ICC/No. 169, respectively (ICC Standard).

Dough mixing properties were determined with a 10-g Mixograph (National Manufacturing Co., Lincoln NE) based on the AACC method 54–40A. Mixing was carried out in triplicate. In this study, eleven Mixograph parameters obtained from the Mixsmart software version 3.8 (AEW Consulting, Lincoln, NE, commercially available through National Manufacturing Division of TMCO, Lincoln NE, USA) were used: eight parameters described the height and width of curves (midline left value, MLV; midline peak value, MPV; midline right value, MRV; midline value at 8 min, MTxV; midline left width, MLW; midline peak width, MPW; midline right width, MRW; midline width at 8 min, MTxW), midline peak time (MPT), midline integral at 8 min (MTxI) and Weakening slope (WS, the difference of MPV and MTxV, indicating mixing tolerance).

### Statistics Analysis

Data were analyzed using the SPSS version 11.0 statistical software package (SPSS Inc., Chicago, Illinois, USA). The general analysis of variance and the least significant difference comparisons of means were used to determine significant difference. The statistical significance for mixing parameters from transgenic lines, non-transgenic line and non-transformed control line was determined using Student’s *t* test.

### Ethics Statement

The described filed studies were approved according to the document ‘The Biosafety Permit of Transgenic Plant Research: The Permit for Field Trial of Transgenic Wheat (No. 033)’, authorized by the Ministry of Agriculture of the People’s Republic of China.

## Results

### Stable Integration of *avenin-like b* Gene in Wheat Plants

In this study, the sequence of *avenin-like b* gene was 855 bp long (GenBank accession number HM027637) and encoded a protein with 284 amino acid residues containing 18 cysteine residues. This predicted protein belonged to Avenin-like b (typ-b3) protein according to the results reported by Kan et al. [22]. The first 18 amino acid residues corresponded to the signal peptide and the mature protein with 266 amino acid residues had a predicted molecular mass of 30 kDa [24]. The phylogenetic relationships of Avenin-like b proteins were analyzed in our previous study [29]. Immature scutella from the wheat cultivar Emai 12 were used as targets for transformation by particle bombardment. Both transgene Avenin-like b proteins from cv. Zhengmai 9023 (GenBank accession number: HM027637) and endogenous Avenin-like b proteins from cv. Emai 12 (GenBank accession number: HM027635) contained 18 cysteine residues (Figure S1). Moreover, the positions of all cysteine residues in both Avenin-like b proteins were consistent with an exception of one nucleotide (Figure S1). Plasmid pAHC25 conferring bialaphos resistance and plasmid pLRPT-avel containing the target *avenin-like b* gene were used for transformation of the wheat variety Emai 12.

**Figure 1:**
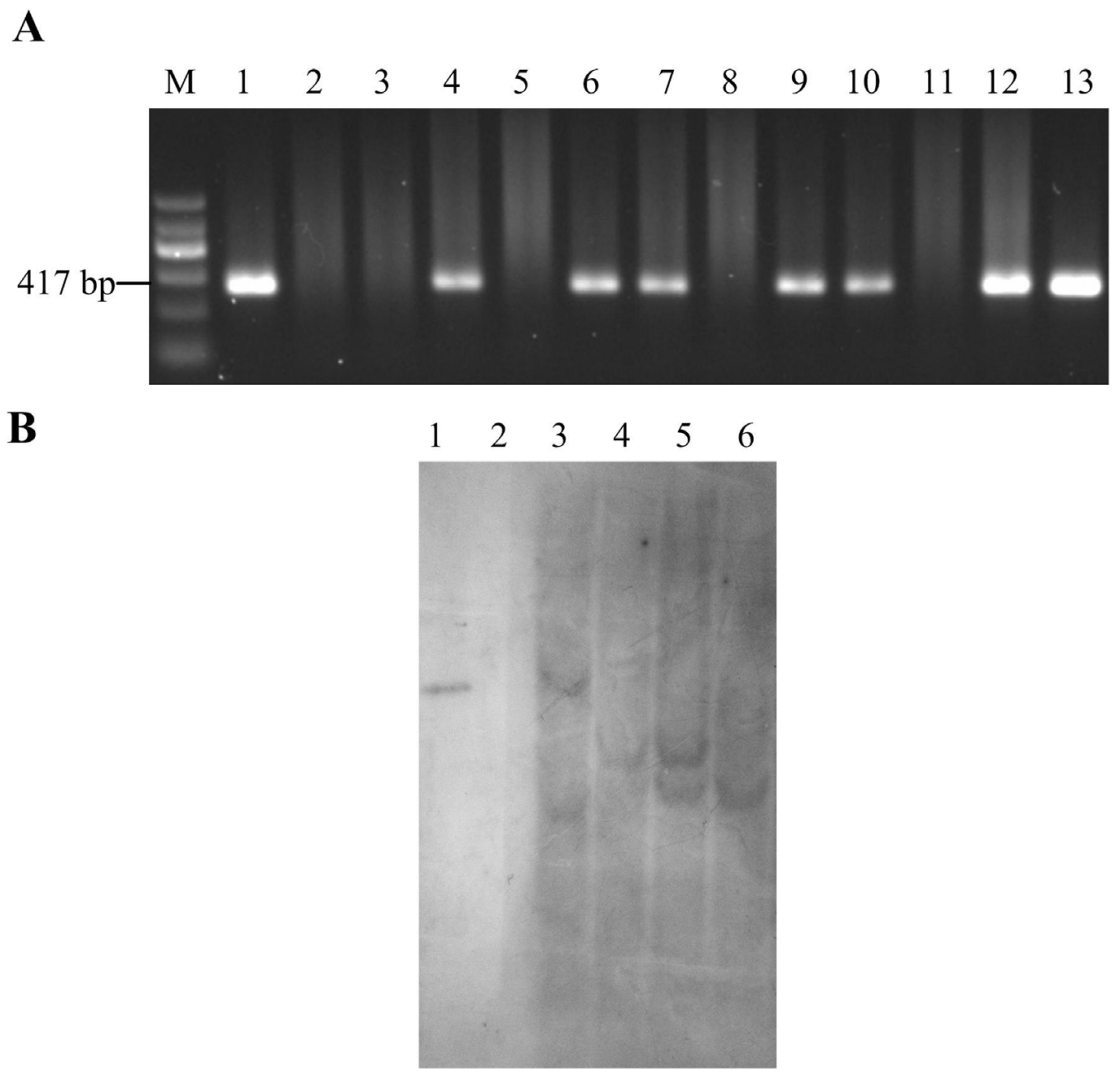
PCR (A) and Southern blotting analysis (B) of the transgenic plants. A. PCR amplification of CaMV35S terminator sequence. Lane M: DNA Marker II; lane 1: plasmid pLRPT-avel for positive control; lane 2: Water for negative control; lane 3: DNA of Emai 12 for negative control; lane 4–13: DNA of regenerated plants. B. Southern blotting analysis of the transgenic wheat lines. Lane 1: Positive control of pLRPT-avel digested with *Bam*HI; lane 2: genomic DNA of Emai 12 digested with *Bam*HI and *Hin*dIII; lane 3–6: genomic DNA of T_0_ transgenic wheat lines digested with *Bam*HI and *Hin*dIII. The PCR results of lane 3–6 in Fig. 1B were shown in lane 6, lane 9, lane 12, and lane 13 of Fig. 1A, respectively.

Transgenic T_0_ plants were confirmed to be positive plants by PCR amplification of the CaMV35S terminator sequence (Figure 1A). Several positive transgenic T_0_ plants resulted from the particle bombardment of about 1050 bombarded immature Emai 12 scutella. The offspring of some transgenic T_0_ plants were further tested and confirmed by Southern blotting and Western blotting analysis.

Southern blotting analysis showed that the selected T_0_ transgenic wheat lines contained single or two bands on the blot (Figure 1B) indicating one or two insertions respectively. The banding patterns, however, were different among the transgenic lines, confirming that the plants were derived from independent transformation events and could therefore be considered as independent lines.

After analyzing five generations (T_0_–T_4_) for the presence and expression of transgene by PCR, SDS-PAGE and Western blotting analysis, two homozygous transgenic wheat lines in T_4_ generation overexpressing *avenin-like b* gene were obtained while the other tested transgenic lines were hemizygous. Two T_4_ homozygous transgenic plant lines (designated E-3 and E-5) together with the non-transgenic line (designated N-1) derived from T_1_ hemizygous plants and non-transformed control Emai 12 plants were grown in the experimental field of the Chinese National Center of Plant Gene Research (Wuhan) HUST Part (Wuhan, Hubei Province, China). The E-3 and E-5 transgenic lines were derived from the T_0_ plants in lane 3 and lane 5 (Figure 1B), respectively. T_5_ seeds were harvested separately from each transgenic line and analyzed by PCR, SDS-PAGE and Western blotting analysis to verify homozygosity and stability of transgene expression. Seeds from the two transgenic lines, one non-transgenic control line and one non-transformed control line were separately harvested and used for all subsequent analyses such as SDSS test and Mixograph analysis.

### Detection of Avenin-like b Protein in Seeds of Transgenic and Control Plants

The pET-32a-avel vector containing the coding region of *avenin-like b* gene, without the signal peptide sequence, was transformed into *E. coli* BL21 (DE3) and induced with isopropyl-β-D-Thiogalactoside (IPTG) for recombinant protein expression. The proteins were then purified by affinity chromatography using His-columns because the recombinant protein contained a His-tag protein at the N-terminus. SDS-PAGE of total proteins extracted from the induced cells showed the expressed recombinant protein to be 50 kDa along with the 20 kDa trx A protein and His-Tag protein fused at the N-terminus [38] (Figure S2). Therefore, the size of the expressed Avenin-like b protein was predicted to be 30 kDa, which was consistent with the molecular mass of the mature Avenin-like b protein encoded by the *avenin-like b* gene. Polyclonal antibodies raised against the renatured heterologously expressed Avenin-like b proteins were also specific to Avenin-like b proteins in the wheat seeds as analyzed by Western blotting analysis (Figure S3).

**Figure 2:**
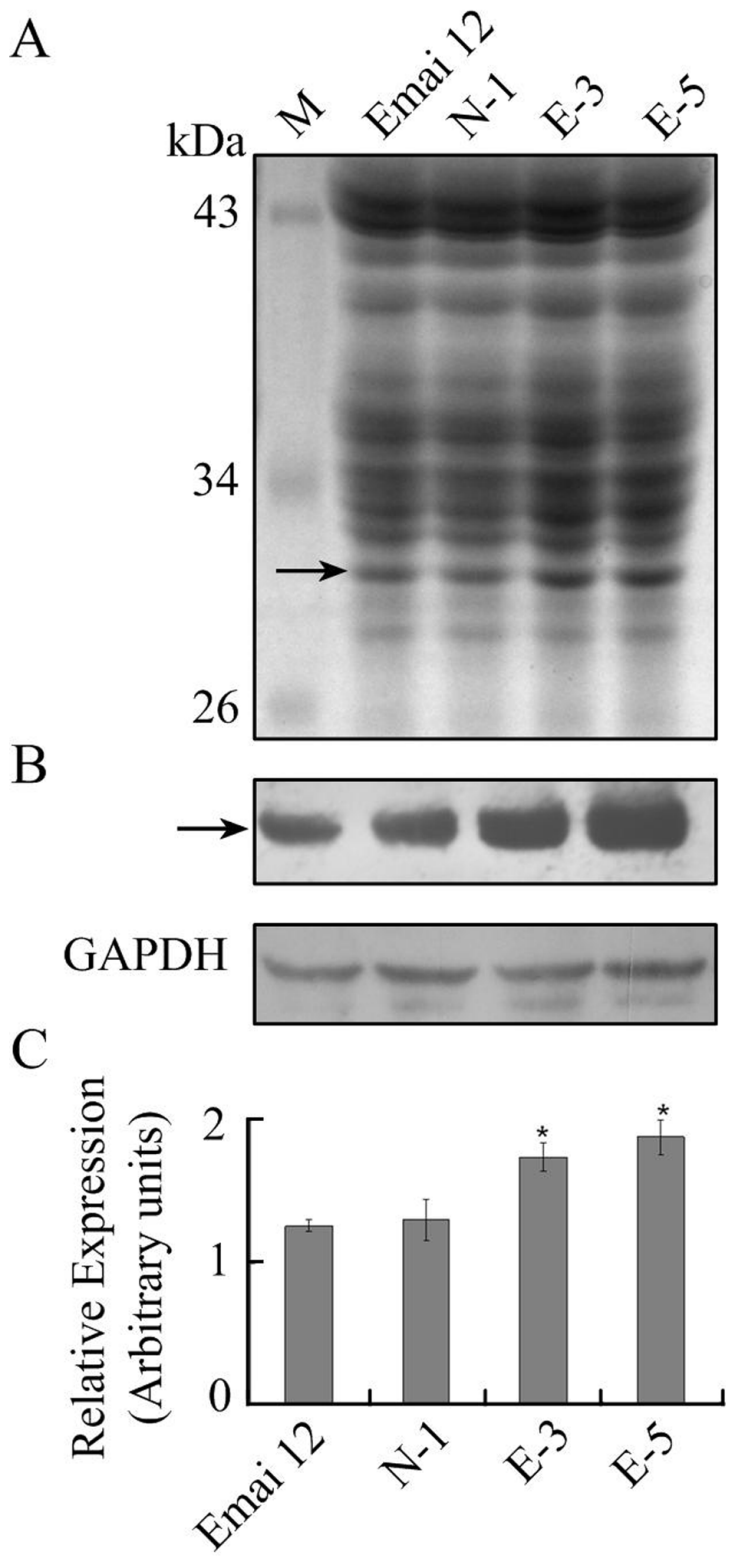
SDS-PAGE and Western blotting analysis of Avenin-like b proteins in transgenic and control lines. Total gluten protein extracted from flours were visualized on stained SDS-PAGE gels (A) and then specifically detected by Western blotting (B). Relative amounts of Avenin-like b proteins (C) were densitometry quantified with respect to the non-transformed control cv. Emai 12. Lines E-3 and E-5 were transgenic lines overexpressing *avenin-like b* gene, while lines N-1 and Emai 12 were non-transgenic line and non-transformed control line, respectively. Arrow indicates the position of the transgenic Avenin-like b proteins. Housekeeping protein GAPDH was used as control to normalize for equal amounts of proteins and to calculate the relative loading volume for each sample.

To investigate the levels of Avenin-like b proteins in the transgenic and control lines, total proteins were extracted and fractionated on SDS-PAGE, followed by Western blotting analysis (Figure 2A and 2B). Staining of the total proteins separated on SDS-PAGE with Coomassie Brilliant Blue allowed the identification of transgenic subunits in wheat lines E-3 and E-5 overexpressing *avenin-like b* gene (Figure 2A). Because of the high sensitivity of Western blotting analysis, it was possible to more accurately identify the Avenin-like b proteins expressed in the wheat grain. In our study, a clear reactive band of the expected molecular mass (about 30 kDa) was observed in all seed protein extracts (Figure 2B). The relative amounts of Avenin-like b proteins in the transgenic wheat lines were measured by using GAPDH as control to normalize for equal amounts of proteins and to calculate the relative amount. These amounts were then compared to the non-transformed control lines. In the transgenic E-3 and E-5 lines, the relative amounts of Avenin-like b proteins increased to 1.46- and 1.53-times, respectively when compared to the non-transformed line, as calculated by densitometry (Figure 2C). However, significant differences were not observed in the amounts of Avenin-like b proteins expressed between the non-transgenic line (N-1) and non-transformed control line (Emai 12) (Figure 2). These results demonstrated that the process of genetic transformation did not alter the amounts of Avenin-like b proteins in line N-1.

### Comparison of Glutenins and Gliadins Amounts between Transgenic and Control Lines

**Figure 3:**
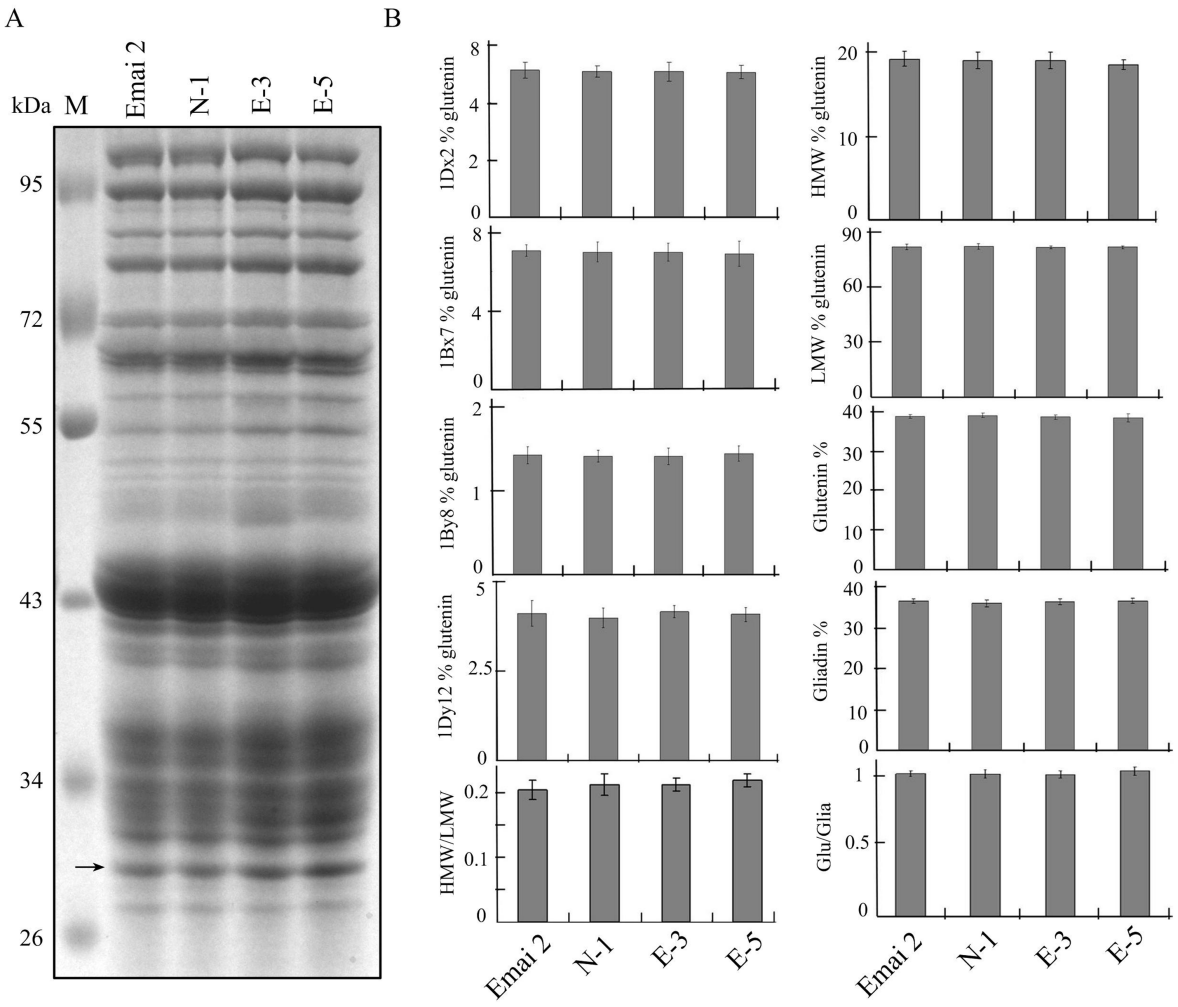
Characterization of storage proteins in transgenic and control lines. A. SDS-PAGE of seed protein extracts from transgenic lines, non-transgenic line and non-transformed control line. Arrow indicates the position of the transgenic Avenin-like b proteins. B. Characterization of storage proteins from the transgenic and control lines. HMW % glutenin and LMW % glutenin mean quantities of HMW-GS and LMW-GS, respectively, expressed relative to total quantity of the glutenins (and the same for 1Dx2%, 1Bx7%, 1By8% and 1Dy12%). HMW/LMW: ratio of the high and low molecular weight glutenin subunits. Glutenin%: quantity of the glutenins expressed relative to total proteins extracted by the sequential extraction methods (and the same for Gliadin %). Glu/Glia: ratio of the glutenins and gliadins. Data are given as mean ± SEM. Values within the same characteristics of storage proteins are not significantly different (P = 0.05).

Comparison of the flour protein amounts between the transgenic and control lines showed variation from 12.3% in Emai 12 control line to 13.4% in the E-5 line (Table 1). Although flour protein amounts in the transgenic lines were higher than those in the control lines, this increase was not statistically significant as determined by analysis of variance (ANOVA). Further, SDS-PAGE results of total storage protein did not show evident difference in their expression patterns among transgenic and control lines (Figure 3A).

**Table 1:** Comparisons of flour quality-related parameters of the transgenic and control wheat lines.

Parameters	Lines			
	Emai 12	N-1	E-3	E-5
*protein characterization*				
Flour protein content (%)	12.5	12.7	13.2	13.4
Sedimentation (ml) at 14%	32.51	32.32	36.67^**^	37.33^**^
Wet gluten (%)	27.23	27.43	33.1^**^	34.9^**^
Dry gluten (%)	8.93	9.02	11.87^**^	11.23^**^
*SE-HPLC*				
%UPP	25.61	24.96	34.35^**^	35.08^**^
*Mixograph*				
Midline left value (% Torque)	34.76	33.9	43.95^**^	42.98^**^
Midline left width (% Torque)	17.29	18.18	19.41^*^	22.08^**^
Midline peak value (% Torque)	38.81	39.2	45.19^**^	45.52^**^
Midline peak width (% Torque)	12.04	11.85	15.74^**^	16.25^**^
Midline peak time (min)	2.85	2.81	3.62^**^	3.74^**^
Midline right value (% Torque)	34.76	33.9	43.95^**^	42.98^**^
Midline right width (% Torque)	9.3	8.6	10.95^*^	12.54^**^
Midline value at 8 min (% Torque)	29.17	28.03	35.7 ^**^	37.02^**^
Midline width at 8 min (% Torque)	4.53	3.96	5.88^ *^	7.52^**^
Midline integral at 8 min(% Torque* min)	270.1	260.37	318.7^**^	312.16^**^
Weakening slope (% Torque)	10.54	10.73	9.25^*^	7.74^**^

Protein contents determined by near-infrared reflectance spectroscopy (NIRS) method were adjusted to a 14% moisture basis.

(*) Means are significantly different to control as determined by the least significant difference comparisons at P<0.05.

(**) Means are significantly different to control as determined by the least significant difference comparisons at P<0.01.

To compare the protein compositions between flour samples from the transgenic and control lines, we separated and quantified glutenins and gliadins. The Glutenin % (quantity of the glutenins expressed relative to total proteins) varied slightly from 38.6% to 38.2% in line E-5 and Emai 12 control, while Gliadin % (quantity of the gliadins expressed relative to total proteins) was very similar in all lines (36.7% in E-5 and 36.5% in Emai 12) (Figure 3B). Neither Glutenin % nor Gliadin % were significantly different between transgenic and control wheat lines as determined by ANOVA. SDS-PAGE analysis of total proteins showed no marked difference in the endogenous HMW-GS (1Dx2, 1Bx7, 1By8 and 1Dy12) subunits between the transgenic and control wheat lines. The proportions of HMW-GS in the glutenin varied from 18.7% in line E-5 to 19.1% in the Emai 12 control line, while those of LMW-GS ranged from 81.1% to 80.9% respectively. Furthermore, the ratios of HMW/LMW were similar among transgenic wheat lines (about 0.21) and control lines (about 0.22). In general, overexpression of Avenin-like b proteins in the transgenic lines did not alter the amounts and proportions of both HMW-GS and LMW-GS glutenins and gliadins in transgenic wheat endosperms.

### Incorporation of the Avenin-like b Proteins into the Glutenin Polymers

**Figure 4:**
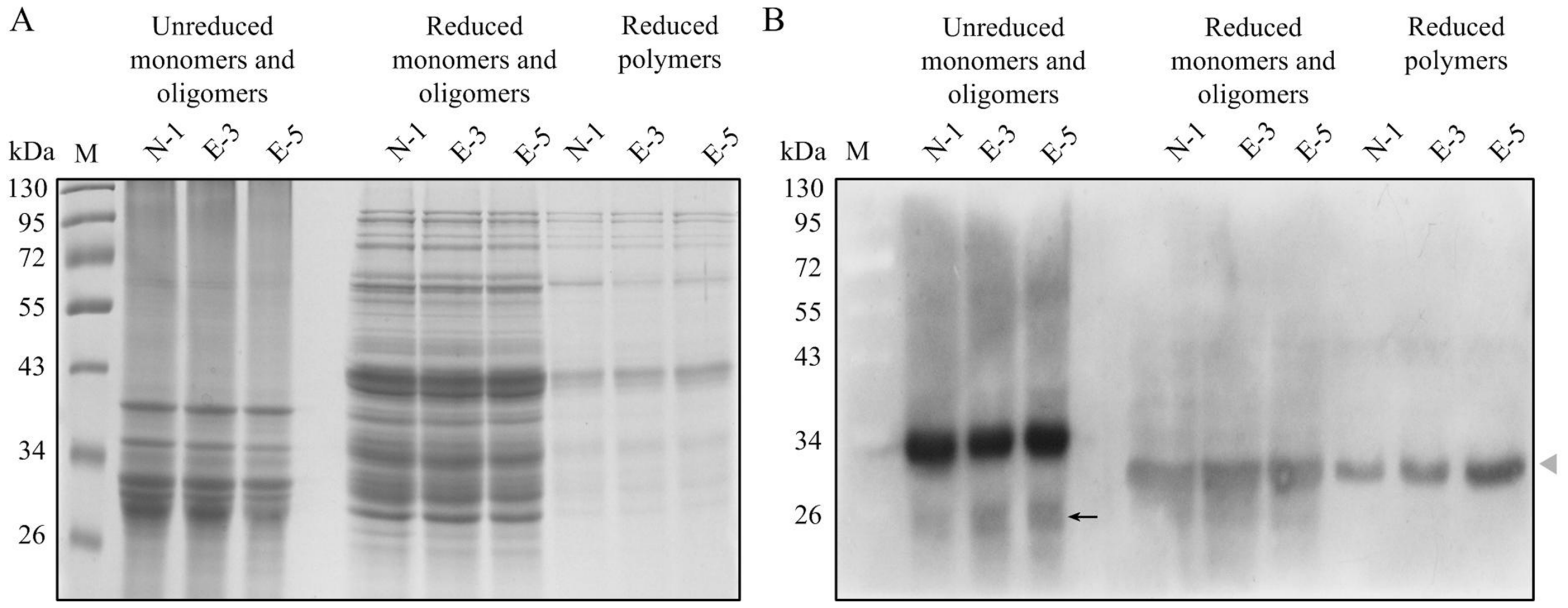
SDS-PAGE and Western blotting analysis of monomeric and polymer fractions in transgenic and control lines. A. SDS-PAGE analysis of reduced and unreduced fractions from flour of non-transgenic control (N-1) line, E-3 transgenic and E-5 transgenic lines. Lane M: Protein Marker. B. Western blotting analysis of reduced and unreduced fraction from flour of non-transgenic control (N-1) line, E-3 transgenic and E-5 transgenic lines. Lane M: Protein Marker. The black arrow (B) indicates the unreduced monomeric subunit, while the grey arrow (B) indicates the reduced form. The unreduced form of the subunit migrates faster probably due to its more compact conformation determined by the presence of intra-chain disulphide bonds.

The Avenin-like b proteins, analyzed in this study, contained 18 cysteine residues hypothesized to form inter-chain disulphide bonds thus allowing incorporation into high molecular mass polymers [22]. To confirm this hypothesis, sequential extraction of monomeric and polymeric proteins from the transgenic (E-3 and E-5) and non-transgenic (N-1) lines was carried out as described by Tosi et al. [31]. The soluble fraction extracted with SDS phosphate buffer contains both monomers and oligomers while the insoluble fraction contains only polymers [31]. Equal amounts of reduced and unreduced extracts were loaded on SDS-PAGE and further analyzed by Western blotting. Figure 4A showed no significant differences in the reduced and unreduced extracts of monomers and oligomers, and the reduced extracts of polymers between the transgenic (E-3 and E-5) and non-transgenic (N-1) line.

Western blotting results of unreduced samples showed only trace amounts of monomers (black arrow in Figure 4B), which migrated slightly faster than the reduced polymers. This could be due to its more compact conformation because of the presence of intra-chain disulphide bonds similar to the results reported by Tosi et al. [31]. In addition, comparison of the Western blotting results obtained with unreduced and reduced samples indicated the present of higher amounts of Avenin-like b proteins in oligomers (Figure 4B). Similarly, it was also more abundant in the polymer protein fraction (grey arrow in Figure 4B) compared to the monomers. Therefore, it was evident that Avenin-like b proteins were predominantly present as both oligomers and polymers. The oligomers detected in the unreduced fractions could be a conformation of the Avenin-like b proteins, and could possibly contain other storage proteins. The unreduced oligomers might also have a more compact conformation determined by the presence of intra-chain disulphide bonds. However, intensity of the bands corresponding to the reduced monomers and oligomers decreased when compared to the corresponding unreduced monomers and oligomers. This was most likely because polyclonal antibodies were raised against the renatured Avenin-like b proteins and could recognize the unreduced conformation better than the reduced one.

In our study, significant differences were not observed in the amounts of Avenin-like b proteins in the monomers and oligomers between the non-transgenic (N-1) and transgenic (E-3 and E-5) lines. However, the amounts of Avenin-like b proteins in the polymers were significantly higher in the transgenic (E-3 and E-5) lines compared to the non-transgenic line (Figure 4B). Especially in transgenic line E-5, Avenin-like b proteins in the polymers were much more abundant than that in transgenic line E-3. These results indicated that extra Avenin-like b proteins in transgenic wheat lines were presumably incorporated into the polymers.

Effects of the transgenes on the proportions of unextractable polymeric proteins (%UPP) were determined by sequential extraction and SE-HPLC using the method described by Tosi et al. [21]. As shown in Table 1, a drastic difference was observed in the %UPP, which was higher in E-3 (34.35%) and E-5 (35.08%) lines when compared to N-1 (25.61%) and Emai 12 (24.96%) lines. Both transgenic lines had higher %UPP values, indicating higher proportions of glutenin polymers in these lines.

### Dough Mixing Properties

The SDSS test showed no difference between the two control lines (N-1 and Emai 12). However, the average SDSS volume was significantly higher in the two transgenic lines (E-3 and E-5) when compared to the control lines (Table 1). Furthermore, statistically significant increases were observed in the wet gluten and dry gluten amounts in the two transgenic wheat lines (E-3 and E-5) compared to the control lines (Table 1).

**Figure 5:**
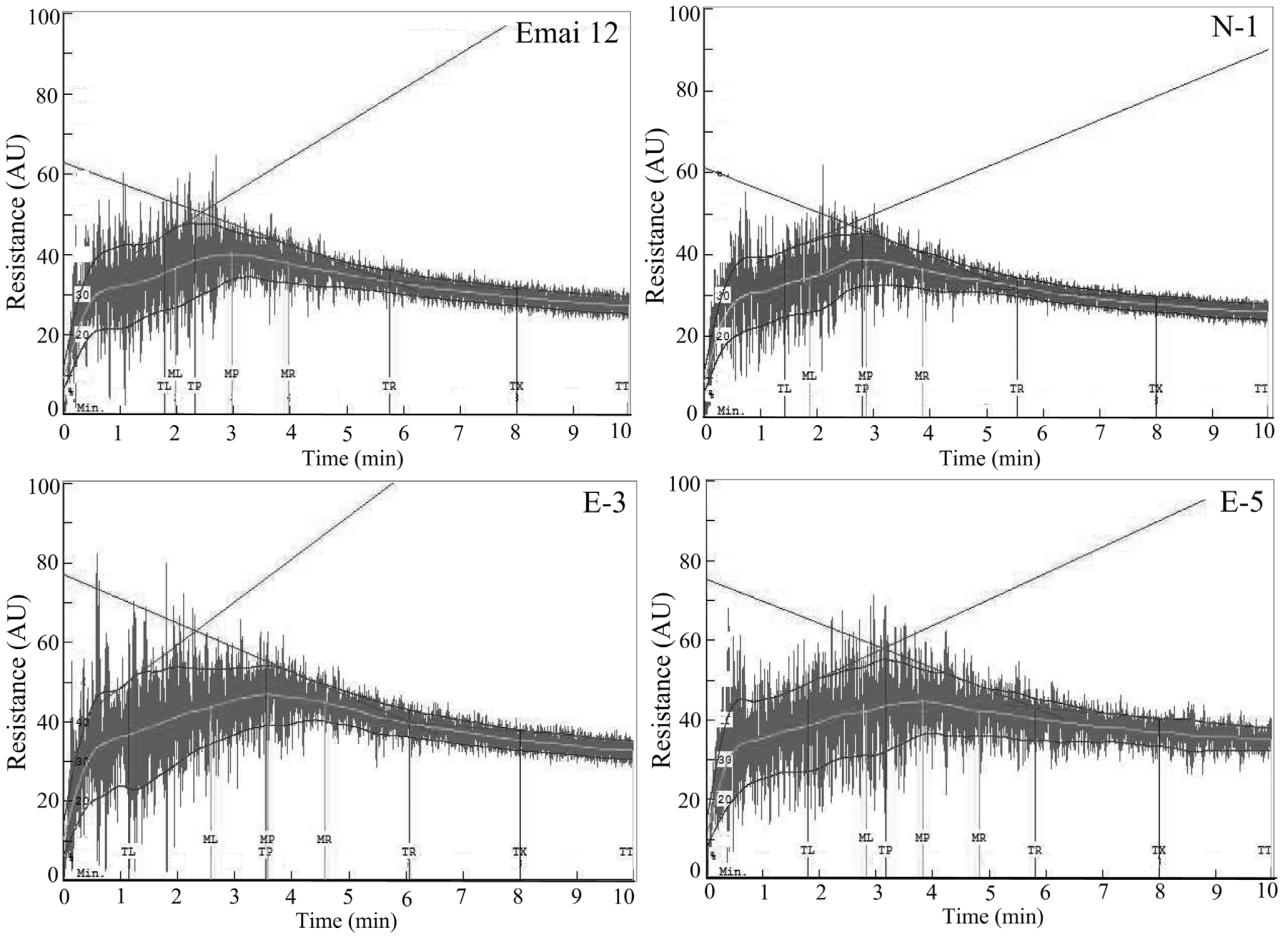
Mixograph curves of dough prepared from transgenic and control lines. Flour samples from two transgenic (E-3 and E-5) lines, one non-transgenic line (N-1) and non-transformed control cv. Emai 12 were subjected to Mixograph analysis to reveal differences in dough mixing properties. (TL: Time before peak of trace envelope. TP: Time to peak of trace envelope. TR: Time after peak of trace envelope. ML: Time before peak of the midline of the trace. MP: Time to peak of the midline of the trace. MR: Time after peak of the midline of the trace. TX: Time at 8 min of mixing. TTT: time at 10 min of mixing.).

The rheological properties of transgenic and control lines were determined using a 10-g Mixograph (Figure 5) and the corresponding parameters were shown in Table 1. No significant differences were observed in the mixing parameters between non-transgenic (N-1) and non-transformed (cv. Emai 12) lines suggesting that particle bombardment and tissue culture of wheat did not affect dough mixing properties (Table 1).

Transgenic lines overexpressing *avenin-like b* gene showed significant increases in the MLV, MPV, MRV and MTxV parameters that corresponded to the heights of curves when compared to the non-transformed control line (Figure 5, Table 1). Heights of the curve after peak resistance remained stable at 43% torque in lines E-3 and E-5, whereas in the Emai 12 it decreased to 34% torque. Moreover, significant increase in bandwidth values (MLW, MPW, MRW and MTxW) was observed in transgenic wheat (E-3 and E-5) lines demonstrating that the dough resistance to extension was improved in transgenic lines overexpressing *avenin-like b* gene. In addition, overexpression of Avenin-like b proteins also reduced the weakening slope (WS). However, this decrease was greater in transgenic line E-5 (7.74%) compared to the control line (10.54%) (Table 1). MPT and MTxI values in both E-3 and E-5 lines were significantly higher than the control lines. MPT in transgenic wheat line E-3 and E-5 increased to 3.62 min and 3.74 min, respectively, compared to 2.85 min in non-transformed wheat lines. More importantly, MTxI values in the E-3 and E-5 lines were also significantly higher compared to control lines, suggesting stronger dough from the flour obtained from E-3 and E-5 lines compared to the control lines.

## Discussion

The aim of this study was to evaluate the effect of Avenin-like b proteins on the functional properties of wheat flour. We have clearly demonstrated that all transgenic lines that tested PCR positive (PCR analysis) for the presence of CaMV35S terminator were also positive for transgene expression, determined using the anti-avenin-like b proteins polyclonal antibody. After selection for four consecutive years, two homozygous transgenic lines overexpressing *avenin-like b* gene (designated E-3 and E-5), one non-transgenic line (designated N-1) and one non-transformed control line (cv. Emai 12) were used to determine the effects of Avenin-like b proteins on dough mixing properties.

### Positive Effects of *avenin-like b* Gene Overexpression on Dough Mixing Properties of Bread Wheat

In this study, we characterized both protein compositions and dough mixing properties (Figure 3 and Figure 5). Because protein compositions played a critical role in dough functionality, we first determined the differences in protein compositions between transgenic and control wheat lines. Amounts of flour protein in the transgenic lines were higher than in control lines (Table 1), although this increase was non-significant. Moreover, differences in the amounts of glutenins and gliadins were observed between transgenic and control wheat lines, but these differences were also not significant (Figure 3A and 3B). The lack of significant difference in protein compositions could be due to the low expression levels of Avenin-like b proteins in the endosperms of bread wheat [22], [23]. Despite an increase in the relative amounts of Avenin-like b proteins in the transgenic E-3 (1.46-times) and E-5 (1.53-times) lines when compared to the non-transformed control line, the proportion of Avenin-like b proteins among the storage protein in transgenic wheat lines was still low compared to glutenins and gliadins (Figure 2 and Figure 3). Therefore, overexpression of Avenin-like b proteins in the transgenic lines did not result in significantly changes in the amounts and proportions of glutenins (including both HMW-GS and LMW-GS) and gliadins in the transgenic wheat endosperms.

**Figure 6:**
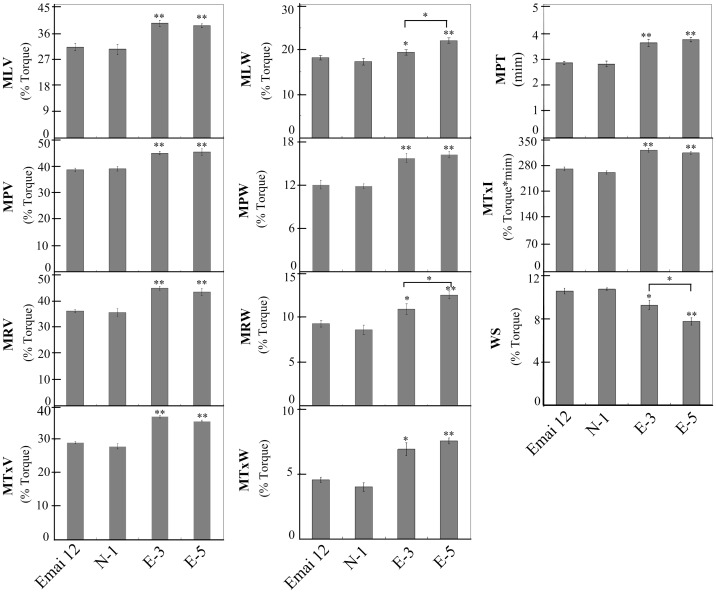
Effect of Avenin-like b proteins on dough mixing properties. Eleven mixing parameters were compared by Student’s *t* test between transgenic lines (lines E-3 and E-5), non-transgenic line (N-1) and non-transformed control line (cv. Emai 12). Data are given as mean ± SEM. * and ** indicates the significant differences with mixing parameters of non-transformed control line at 0.05 or 0.01 probability level, respectively.

The SDSS test and Mixograph are small-scale tests used to analyze dough gluten strength and mixing properties [39], [40], [41], which showed significant increases in the average SDSS volume observed in the transgenic lines (Table 1). These values showed that the transgenic lines had better quality than the control lines. High SDSS volumes have been shown to be associated with stronger gluten and superior bread-making quality [39], [42]. Mixing properties of transgenic lines analyzed using the 10-g Mixograph provided information on dough strength, which closely correlated with baking quality. Relationships between Mixograph parameters and dough viscoelasticity have been studied in details [40] and associations of these parameters with other wheat quality traits were also discussed previously [43]. In general, MPW and MRW positively correlated with dough resistance to extension [21], whereas WS and MTxW negatively and positively correlated with tolerance to over-mixing, respectively [40], [44], [45]. Parameters of the curve height values (MLV, MPV, MRV and MTxV) positively correlated with dough elasticity [39]. MPT, MPV and MTxI also positively correlated with dough strength [17], [45]. In general, weaker dough has higher WS, shorter MPT, lower MPV and smaller MTxI when compared to stronger dough [17], [45]. In our study, significant differences in mixing parameters were not observed between the non-transgenic (N-1) line and non-transformed wheat control line (Figure 6) demonstrating the nearly identical patterns of storage proteins in N-1 line when compared to the transformed control line (Figure 3). This result met the expectation that the genetic transformation process did not caused any variations in storage proteins in N-1 line.

We further compared the mixing parameters between transgenic lines (lines E-3 and E-5), non-transgenic line (N-1) and non-transformed control line (cv. Emai 12) (Figure 6). In transgenic lines, a significant increase in all parameters related to the curve heights demonstrated an increase in dough elasticity. Significantly lower WS values and higher MTxW values in E-3 and E-5 lines revealed an improved mixing tolerance because of the overexpression of Avenin-like b proteins. In addition, a significant increase in all mixing parameters related to curve widths in the transgenic lines when compared to control lines demonstrated that dough resistance to extension was also improved. Further, increase in MPT, MPV and MTxI in lines E-3 and E-5 indicated the enhancement of dough strength in the transgenic lines in comparison with the control lines. From these results, it could be concluded that overexpression of Avenin-like b proteins generally led to improve dough elasticity, mixing tolerance and dough resistance to extension. These results were also consistent with our previous report that the direct addition of Avenin-like b proteins into flour resulted in statistically significant increases in peak resistance and mixing tolerance in an *in vitro* assay [29], indicating that Avenin-like b proteins had advantageous effects on mixing properties of wheat flour.

In addition, significant differences were observed in MLW, MRW and WS between the two transgenic wheat lines (Figure 6). These differences had quantity effect on the dough quality because of the different expression levels of Avenin-like b proteins in the two transgenic wheat lines. These results were consistent with the results of Chen et al. [29] who reported that the incorporation 15 mg Avenin-like b proteins into the flour had larger effect on mixing properties than 10 mg as shown *in vitro*. Both *in vitro* and *in vivo* studies suggested that the effect of Avenin-like b protein on dough quality could be enhanced with an increase in the quantity of this protein.

### Incorporation of Transgenic Subunit into the Glutenin Polymers

Both the amount and size distribution of polymeric proteins have been shown to be important and their contributions to baking performance have been extensively studied and documented throughout the world [8], [13], [37], [46]. Moreover, a certain amount of these polymers remains unextractable (UPP) in various extracting systems (e.g. SDS phosphate buffer or acetic acid solution) and the proportion of the unextractable polymeric protein (%UPP) fraction is reported to be related to the rheological properties of wheat flour [37], [39].

The Avenin-like b proteins, analyzed in this study, contained 18 cysteine residues speculated to form intra-chain and inter-chain disulphide bonds indicating that they could be integrated into glutenin polymers to alter the functional properties of wheat flour [22]. To confirm this hypothesis, monomeric subunits, small oligomers and polymers were extracted and analyzed which showed that Avenin-like b proteins existed predominantly in both oligomer and polymer proteins rather than in monomeric fraction. Moreover, the amounts of Avenin-like b proteins in the polymers were higher in the transgenic (E-3 and E-5) lines compared to the non-transgenic line (Figure 4). Based on these results, it was reasonable to conclude that in transgenic plants Avenin-like b proteins were indeed incorporated into the glutenin polymers. This observation was also supported by the high proportion of unextractable polymeric protein (%UPP), which represents a way to measure polymer size distribution, in the transgenic wheat lines (Table 1). These results demonstrated that the improvement of flour properties in the transgenic wheat lines were associated with an increased proportion of polymer proteins. It could be postulated that the overexpressed Avenin-like b proteins can affect the formation of endogenous protein network and impact on the cross-linking of glutenin monomers *via* inter-chain disulphide bonds [22].

Additionally, in cereals, the formation of disulfide bonds appears to increase structural stability of the folded conformation and to decrease solubility of storage proteins [47], [48]. Both of these features provide protection against proteolysis which might be related to seed dormancy [47], [48]. The biological role of storage proteins, in fact, is to act as a source of carbon, nitrogen and sulphur for germination and seedling growth through storage proteolysis in cereal plants [48], [49], [50]. When conditions are favourable for germination, the disulfide bonds of storage proteins are reduced by a regulatory disulfide protein Thioredoxin which could reduce the disulfide bonds of storage proteins, thereby increasing the susceptibility to proteolysis of storage proteins and facilitating germination [49], [50], [51], [52], [53]. As discussed above, overexpression of Avenin-like b proteins could significantly affect on the proportion of polymeric gluten proteins by inter-chain disulphide bonds and might have a function for seed physiology during dormancy and germination.

This is the first report on producing transgenic lines overexpressing *avenin-like b* gene and the first evidence for the positive effect of Avenin-like b proteins on dough mixing properties of wheat. This study is also the first to confirm the incorporation of Avenin-like b proteins into the gluten polymers in wheat by inter-chain disulphide bonds. These results essentially extended our knowledge of the role of Avenin-like b proteins in determining the functionality of bread wheat, which should help understand the influence and mechanism of Avenin-like b proteins on the quality of wheat flour. The *avenin-ike b* gene could be an excellent candidate gene to improve the functional properties of wheat. It would be of interest to determine whether the Avenin-like b proteins with different compositions of gluten proteins or in different genotypes could have different effects on the functional properties of dough, and whether the variation in the number and/or the positions of cysteine residues in Avenin-like b proteins could have different effect on mixing properties of dough. Additional studies are required to better understand the role of Avenin-like b proteins in functional properties of wheat flour and their possible biological meaning (e.g. role in grain development and seed germination).

## Supporting Information

Figure S1 **Alignment of the amino acid sequences of Avenin-like b proteins from wheat.** The asterisks indicate the cysteine residues and their locations, while the black arrow indicates the different nucleotide between transgene Avenin-like b proteins from cv. Zhengmai 9023 (GenBank accession number: HM027637) and endogenous Avenin-like b proteins from cv. Emai 12 (GenBank accession number: HM027635).
(TIF)

Figure S2 **Expression and purification of Avenin-like b protein from** ***E. coli*** **.** Lane M: Protein Marker; lane 1: Purified Avenin-like b protein (with His tag); lane 2: Proteins extracted from *E.coli* BL21 (DE3) transformed with pET-32a-avel after addition of IPTG; lane 3: Proteins extracted from *E.coli* BL21 (DE3) transformed with pET-32a-avel without addition of IPTG.
(TIF)

Figure S3 **Polyclonal antibodies were specific to Avenin-like b proteins as analyzed by Western blotting.** Lines E-3 and E-5 were transgenic lines overexpressing *avenin-like b* gene, while lines N-1 and Emai 12 were non-transgenic line and non-transformed control line, respectively. Arrow indicates the position of the Avenin-like b proteins. Lane M: Protein Marker.
(TIF)
