# Supplementary figures and images for: Overexpression of Avenin-Like b Proteins in Bread Wheat (Triticum aestivum L.) Improves Dough Mixing Properties by Their Incorporation into Glutenin Polymers
Source: PLoS One. 2013 Jul 2;8(7):e66758. doi: 10.1371/journal.pone.0066758 (PMC3699606; doi:10.1371/journal.pone.0066758)

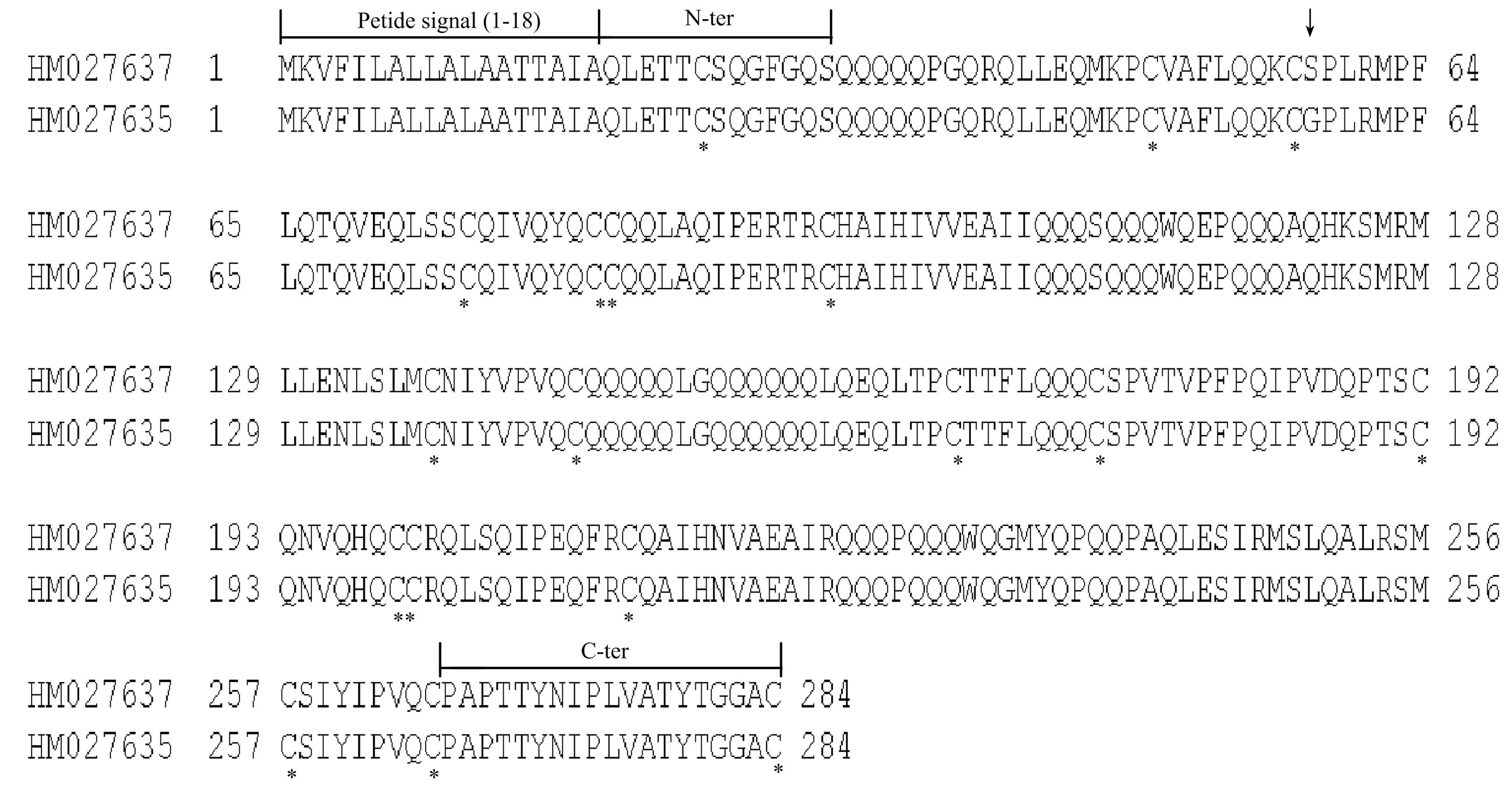

Supplement: Figure S1 — Alignment of the amino acid sequences of Avenin-like b proteins from wheat. The asterisks indicate the cysteine residues and their locations, while the black arrow indicates the different nucleotide between transgene Avenin-like b proteins from cv. Zhengmai 9023 (GenBank accession number: HM027637) and endogenous Avenin-like b proteins from cv. Emai 12 (GenBank accession number: HM027635). (TIF) [file pone.0066758.s001.tif]

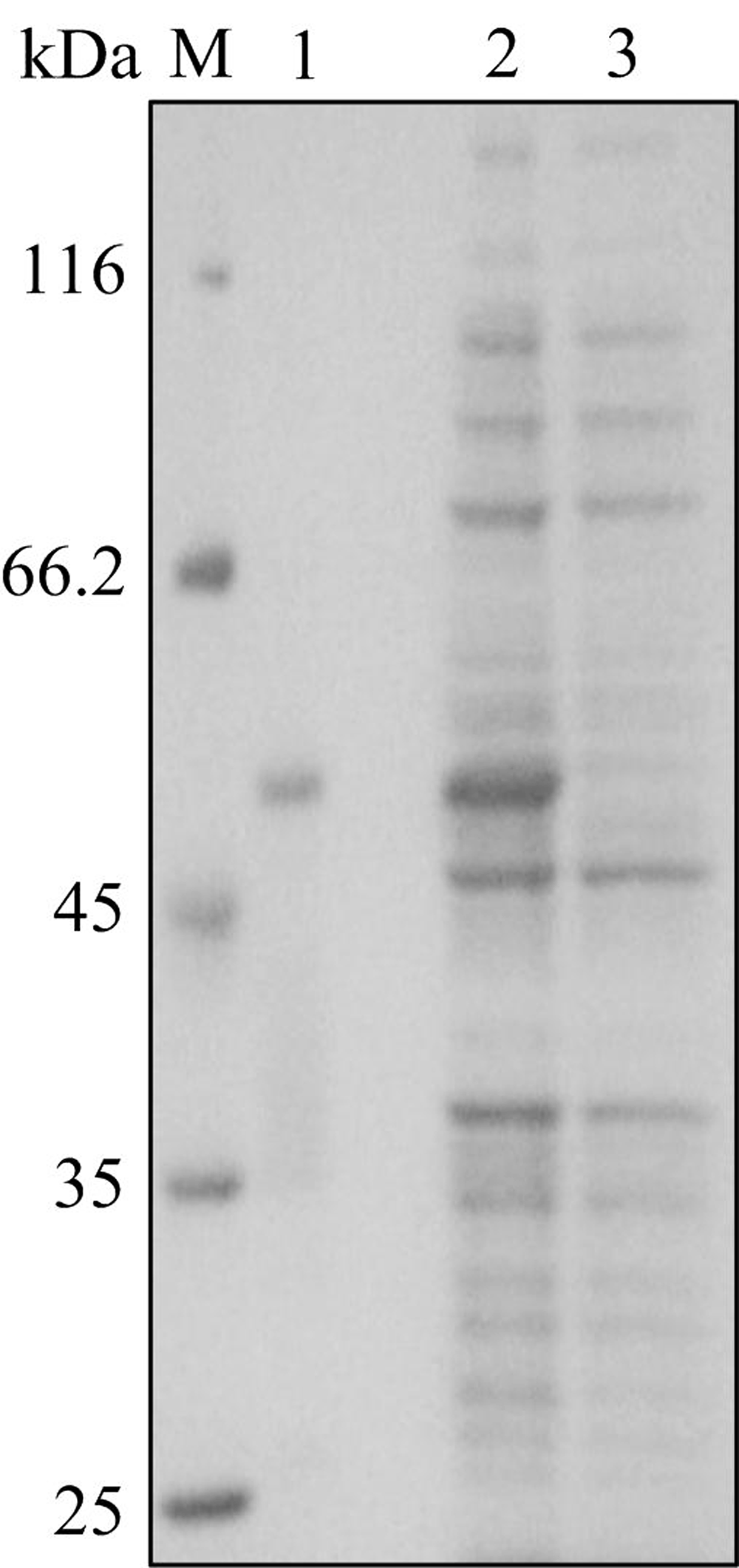

Supplement: Figure S2 — Expression and purification of Avenin-like b protein from E. coli . Lane M: Protein Marker; lane 1: Purified Avenin-like b protein (with His tag); lane 2: Proteins extracted from E.coli BL21 (DE3) transformed with pET-32a-avel after addition of IPTG; lane 3: Proteins extracted from E.coli BL21 (DE3) transformed with pET-32a-avel without addition of IPTG. (TIF) [file pone.0066758.s002.tif]

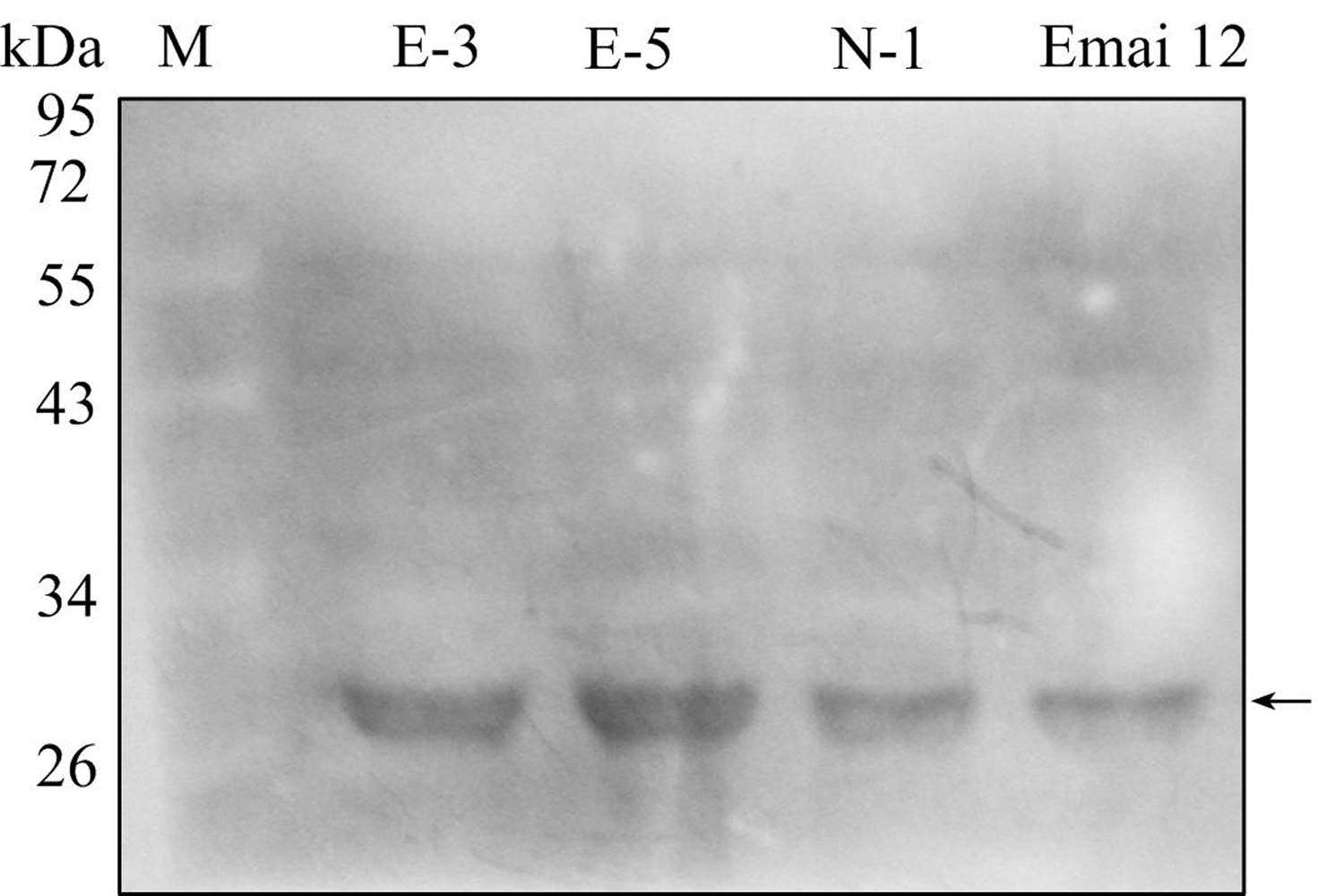

Supplement: Figure S3 — Polyclonal antibodies were specific to Avenin-like b proteins as analyzed by Western blotting. Lines E-3 and E-5 were transgenic lines overexpressing avenin-like b gene, while lines N-1 and Emai 12 were non-transgenic line and non-transformed control line, respectively. Arrow indicates the position of the Avenin-like b proteins. Lane M: Protein Marker. (TIF) [file pone.0066758.s003.tif]
